# Gas-Phase Electrophoresis (nES GEMMA Instrumentation) of SARS-CoV-2-Based Virus-like Particles

**DOI:** 10.1021/acsomega.5c06369

**Published:** 2025-11-12

**Authors:** Victor U. Weiss, Martina Marchetti-Deschmann

**Affiliations:** Institute of Chemical Technologies and Analytics, TU Wien, Getreidemarkt 9/164 CTA, 1130 Vienna, Austria

## Abstract

Gas-phase electrophoresis separates singly charged particles according to their size at ambient pressure in a high laminar sheath flow of particle-free air and a tunable electric field. Subsequent detection of analytes is particle-number-based. Such a setup is very robust and has been successfully applied for several (bio)­nanoparticle-containing materials, e.g., virus-like particles (VLPs). These resemble their parent viruses but due to lack of genomic material are noninfectious. Hence, VLPs find great interest, e.g., in the fields of vaccine development, shielded cargo transport, gene therapy, or parent virus characterization. In all those cases, information on particle size, size distribution, analyte stability, particle numbers, and particle-size derived molecular weight (MW) values yields valuable insights into the VLP in question. Focusing on SARS-CoV-2 VLPs, the source of the recent COVID-19 pandemic, we demonstrate for the first time that gas-phase electrophoresis on a nES GEMMA (nano-electrospray gas-phase electrophoretic mobility molecular analyzer) also known as nES DMA (differential mobility analyzer) or SMPS instrument is possible. VLPs can be detected as broad, size-heterogeneous peaks next to low MW material. Host-cell proteins as well as VLP building blocks can thus be analyzed with the same setup as native macromolecules. Therefore, nES GEMMA measurements are able to support characterization of SARS-CoV-2 VLP-containing samples in terms of VLP stability, particle size, number concentration, and MW values (based on a MW/nES GEMMA-derived particle size correlation).

## Graphical abstract

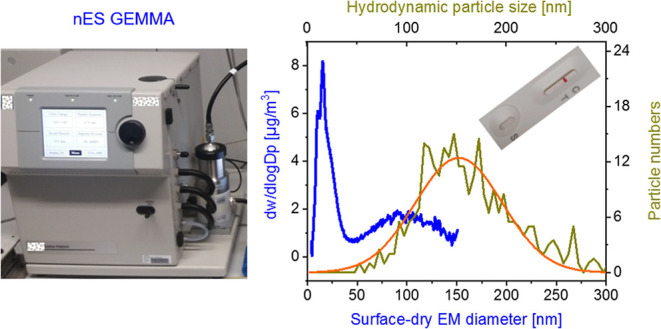

## Introduction

Over the years, gas-phase electrophoresis on a nano-Electrospray Gas-phase Electrophoretic Mobility Molecular Analyzer (nES GEMMA),^1^ also known as nES Differential Mobility Analyzer (nES DMA), MacroIMS, LiquiScan ES, or Scanning Mobility Particle Sizer (SMPS), has shown its ability in (bio)­nanoparticle research. Analytes in the size range from several few to several hundred nanometers in diameter are electrosprayed from a volatile electrolyte solution, for instance, ammonium acetate. Subsequently, droplets dry down and at the same time, charge equilibration in a bipolar atmosphere, induced, e.g., by a radioactive source like ^210^Po,^2,3^ a soft X-ray charger,^4^ or a bipolar corona discharge process,^5^ occurs. Hence, and depending on the size of analytes, a certain percentage of singly charged analytes is obtained.^6^ Usually, multiply charged analytes can be neglected due to their low occurrence. Likewise, neutral particles do not play any further role in the separation process by applying an electric field.

Separation of surface dry analytes occurs in a high-laminar sheath flow of particle-free ambient air and a tunable electric field. By variation of the field strength, particles of different sizes are able to pass through the DMA unit of the instrument according to electrophoresis principles. Following size separation, particles are counted after a nucleation step in a supersaturated atmosphere of either *n*-butanol or water in an ultrafine condensation particle counter (CPC) as they pass a focused laser beam. Relating particle counts to electric field strength values necessary for particles to pass the DMA size filter and ultimately the surface dry particle size (electrophoretic mobility, EM, diameter) finally yields a spectrum based on particle-number concentrations in accordance with recommendations of the European Commission for nanoparticle characterization (2011/696/EU, October 18, 2011, updated version 2022/C 229/01, June 10, 2022).

A corresponding setup has been used in a multitude of studies,^7^ e.g., focusing on the characterization of viruses^8,9^ and virus-like particles (VLPs),^10,11^ liposomes,^12,13^ lipoproteins and extracellular vesicles,^14,15^ proteins and protein aggregates,^16^ carbohydrates,^17^ organic and inorganic nanoparticles,^18^ polymers,^19,20^ and genetic material.^21^

VLPs are biological nanoparticles based on a proteinaceous core and sometimes include an additional lipid envelope. They are based on parent viruses, but in contrast to virus particles, VLPs lack the necessary genomic material for the infection of target cells. Instead, VLPs can be applied, e.g., for vaccination purposes (as they are indistinguishable in their shell from their parent virus), as carrier particles for well-defined genomic information (as applied in the field of gene therapy), or as drug delivery vesicles.^22^ The huge benefit of VLPs over their parent viruses is their noninfectivity. Gas-phase electrophoresis of VLPs enabled i.a. the determination of bionanoparticle size,^11,23,24^ molecular weight (MW),^10,25^ or the thorough characterization of VLP material out of complex samples by combination of nES GEMMA with orthogonal analytical techniques.^26^

Limitations in sample handling due to analyte infectivity when working with viruses are the reason that gas-phase electrophoresis of SARS-CoV-2, the coronavirus of the recent COVID-19 pandemic, to our knowledge has not been carried out so far. However, since recently, SARS-CoV-2-based VLPs have been commercially available. It was therefore the aim of this study to characterize SARS-CoV-2 VLPs via gas-phase electrophoresis on nES GEMMA instrumentation for the first time. So far, only human coronavirus OC43 has been analyzed via a similar setup.^27^

## Materials and Methods

### Chemicals

Ammonium acetate (≥99.99% trace metals basis) and ammonium hydroxide (approximately 28–30% [w/v] ammonia in water, ACS reagent) were obtained from Sigma-Aldrich (St Louis, MO, USA). Water was from a Millipore apparatus and showed 18.2 MΩcm resistivity at 25 °C (Merck, Darmstadt, Germany). SARS-CoV-2 VLPs produced in HEK293 cells were obtained from Leadgene Biomedical Inc. (Tainan City, Taiwan) as a 1 mg/mL solution in phosphate-buffered saline, pH 7.4 (PBS).

### Electrolyte Solution

Aqueous ammonium acetate, 40 mM, pH 8.6, was used as an electrolyte for nES GEMMA measurements. It was filtered through 0.2 μm pore size, cellulose acetate membrane syringe filters (Minisart, Sartorius, obtained via Sigma-Aldrich).

### Instrumentation

Gas-phase electrophoresis was carried out on a nES GEMMA instrument (TSI Inc., Shoreview, MN, USA) consisting of a 3480C nES aerosol generator with a bipolar corona discharge device (MSP 1090), a 3080C classifier with a 3085 nano-DMA, and a 3776C *n*-butanol-driven ultrafine condensation particle counter. MacroIMS manager v2.0.1 was used for instrument control. Particle-free air was additionally dried (Donaldson Variodry Membrane Dryer Superplus obtained via R. Ludvik Industriegeräte, Vienna, Austria) prior to application. For the nES process, a homemade 25 μm inner diameter, cone-tipped fused silica capillary was used.^28^ NTA measurements were taken on a PMX-120-S instrument from Particle Metrix GmbH (Inning, Germany). Zetaview 8.05.05 SP2 was used for instrument control. Clungene SARS-CoV-2 antigen tests (detecting SARS-CoV-2 N protein) were obtained from a local pharmacy.

### Sample Preparation

Thirty microliters of SARS-CoV-2 VLPs in PBS were weighed to 470 μL ammonium acetate on top of a 300 kDa MW cutoff Nanosep filter (Pall, obtained via VWR, Vienna, Austria). Subsequent spinning was at 1500 × *g* until most of the liquid had passed the membrane (approximately 7–9 min). The eluate was discarded, and 500 μL of ammonium acetate was replenished on the filter membrane. Centrifugation and subsequent handling steps were repeated for four overall centrifugation steps. The retentate was recovered and adjusted to 45 μL with ammonium acetate based on the original weighed sample value and the sample weight after spin filtration (in overall 1:1.5 sample dilution). If indicated, a SARS-CoV-2 VLP containing sample further diluted to 50 or 25% (v/v) in ammonium acetate was used.

### Measurement and Data Evaluation

nES GEMMA measurements recorded spectra for 235 s/spectrum and 5 s reset time of the DMA voltage. Seven spectra after nES GEMMA equilibration to a new sample were combined via their medians to obtain data as presented. The nES was run under conditions enabling a stable Taylor cone (typically around 1.85 kV and −380 nA current with 0.1 L/min (Lpm) carbon dioxide and 1.0 Lpm particle-free, pressurized, and additionally dried ambient air). Four pounds per square inch differential (PSID) were applied for sample introduction to the nES capillary. Measurements were recorded at either a 3.5 Lpm sheath flow of particle-free air in the nano-DMA (scan range 4.0–149.9 nm EM diameter) or 15.0 Lpm (scan range 2.0–64.9 nm EM diameter). For Figure 2 also, intermediate settings were applied.

NTA measurements were carried out at “sensitivity: 70”, “frame rate: 30”, “shutter: 100”, “min brightness: 29”, “max. size: 230”, “min size: 5”, and “trace length: 7” settings. Samples were diluted in Millipore grade water (1:800 [v/v]) prior to analysis.

Data was plotted and Gauss peaks fitted in Origin software (OriginPro 2019 (64-bit), v9.6.0.172, OriginLab cooperation, Northampton, MA, USA).

## Results and Discussion

SARS-CoV-2 belongs to the family of coronaviridae and is formed out of a 60–120 nm diameter-sized proteinaceous capsid additionally supported by a lipid envelope. Viral proteins S, N, M, and E form the capsid and protect, in the native viral state, an approximately 30 kbases long RNA genome. S protein aggregates on the viral surface are responsible for the characteristic microscopic appearance of these bionanoparticles showing spikes (https://viralzone.expasy.org/764?outline=all_by_species, retrieved on December 12, 2023). In our manuscript, we worked with a commercially available preparation batch of SARS-CoV-2 VLPs provided by Leadgene Biomedical, Inc., (https://www.leadgenebio.com/sars-cov-2-virus-like-particles-ldg002pvm.html, retrieved on August 7, 2025), which have been characterized by (i) Nanoparticle Tracking Analysis (NTA) yielding a particle concentration of an average of 1.0 × 10^10^ VLP particles per mL and a maximum hydrodynamic particle size of approximately 160 nm, (ii) negative stain transmission electron microscopy (TEM), and (iii) Western blot analysis applying mouse anti-SARS-CoV-2 spike monoclonal and anti-SARS-CoV-2 NP polyclonal antibody.

### Preparation of SARS-CoV-2 VLPs Containing Samples for Gas-Phase Electrophoresis

For a first round of experiments, we focused on preparation of the SARS-CoV-2 VLP sample for gas-phase electrophoresis. In doing so, we opted for spin filtration (300 kDa molecular weight cutoff filters) of VLP solutions at low centrifugal forces (1500 × *g*) to exchange low molecular weight sample material with volatile, aqueous ammonium acetate. Figure 1A demonstrates that this approach did not alter the bionanoparticle composition of the VLP sample in terms of particle size or concentration as assessed via NTA.

**Figure 1:**
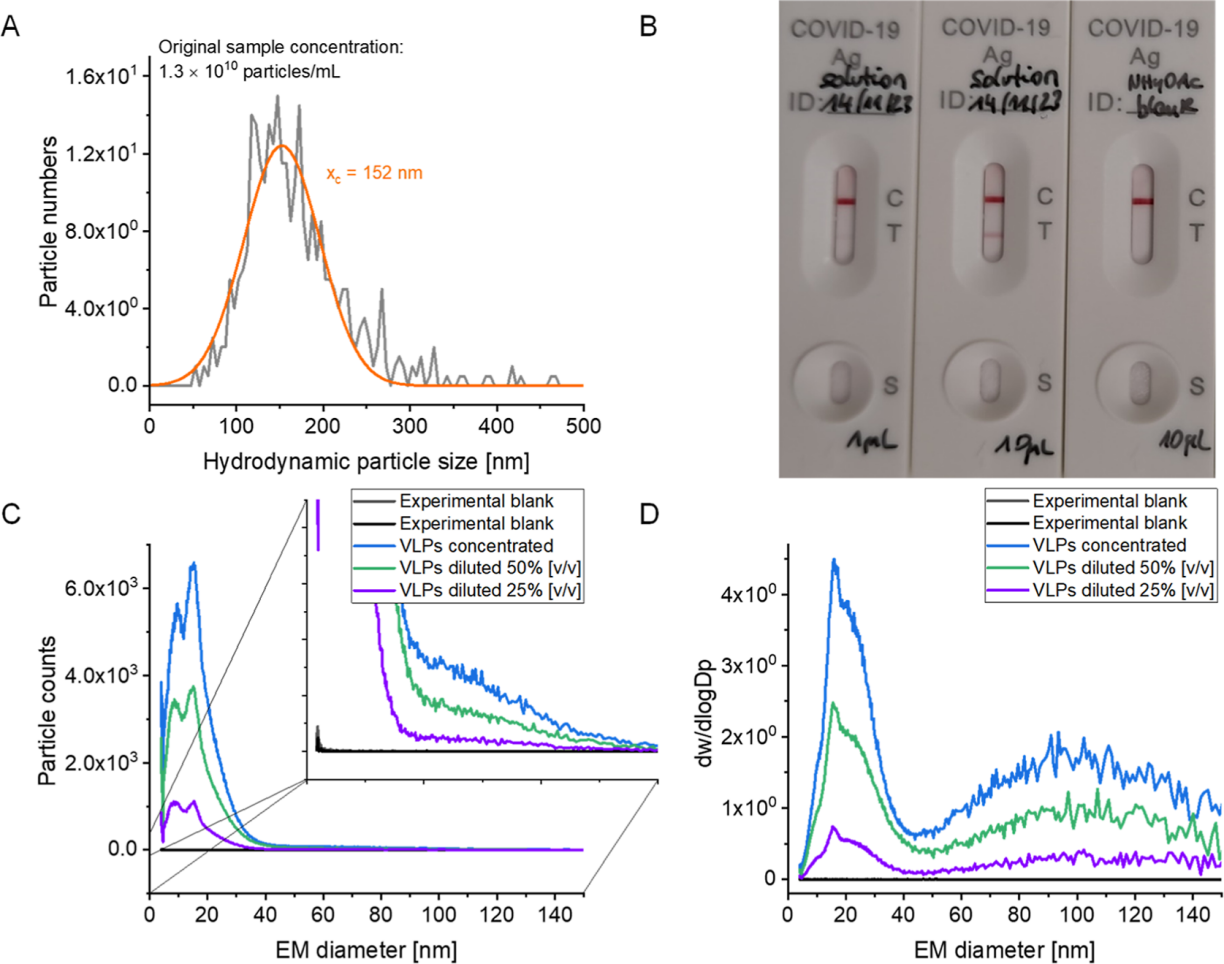
SARS-CoV-2 samples after electrolyte exchange show particle numbers comparable to the original sample (1.0 × 10^10^ particles per mL stated by the manufacturer) as assessed via NTA (average from two measurements). (A) A Gaussian curve (orange) was fitted to measured data (gray). Antigenicity (B) was assessed via a commercially available antigen test targeting SARS-CoV-2 N protein. Two different SARS-CoV-2 sample volumes were mixed with a corresponding antigen extraction buffer (left, middle) and results compared to an electrolyte blank (right). nES GEMMA with particle number (C) and mass-based (D) data evaluation reports particles in the size range from approximately 50 to 150 nm EM diameter next to low molecular weight material up to approximately 30 nm EM diameter.

VLP antigenicity (Figure 1B) was targeted via a commercially available SARS-CoV-2 antigen test kit. A second line at position T indicates antigen, i.e., SARS-CoV-2 N protein, presence. Taking the sample subsequently to the nES GEMMA instrument yields results as shown in Figure 1C for overall particle counts. High particle count values are obtained in the range up to 30 nm EM diameter, followed by a signal of low intensity in the range of interest for VLPs from approximately 50 to 150 nm. It is of note that an experimental blank, plain ammonium acetate treated like a VLP containing sample in terms of filtration, failed to yield corresponding nES GEMMA signals, just displaying baseline values. Also, two dilutions of the initial sample as well as a repeated preparation of the VLP containing sample were investigated. All of these samples reported the initially described signals. From the sum of these investigations, the presence of corresponding VLP analytes and no mere unspecific aggregation of sample components is therefore highly probable.

It is of note that for heterogeneous samples, we usually plot particle count values as reported from the TSI Inc. software despite several other data export options. These latter include additional correction factors, e.g., for multiply charged analytes or detection efficiency. However, previously, we reasoned that in case of very complex samples or heterogeneous analytes, these additional corrections could introduce artifacts to resulting spectra.^17^ Nevertheless, one potential possibility of data evaluation enables mass-based data evaluation, i.e., the display of dw/dlogDp including the aforementioned correction factors. For a steady baseline with only a few occurring low detector events over the whole analysis range, such a display results in an exponentially increasing baseline with increasing EM diameters. However, plotting the same data as presented in Figure 1C accordingly yields a clearly discernibly second peak from 50 to 150 nm EM diameter next to low molecular weight material (Figure 1D). This data is in line with hydrodynamic particle diameter values obtained from NTA measurements for SARS-CoV-2 VLPs and enables better visualization of nES GEMMA results due to overall low particle numbers and a heterogeneous size distribution of analytes.

### Investigation of Low EM Diameter Sample Components

Following our initial nES GEMMA measurements, we took interest in the low MW material detected in VLP samples up to approximately 30 nm EM diameter. It is of note that these sample constituents other than those with gas electrophoresis cannot be detected via NTA measurements due to instrument-inherent limitations. The occurrence of such material is surprising as sample preparation includes a spin filtration step applying a 300 kDa MW cutoff filter membrane (300 kDa MW cutoff corresponds approximately to 11 nm EM diameter on the protein MW scale based on a MW/EM diameter correlation^29^). Clearly, SARS-CoV-2 samples also show material below this EM diameter threshold. Low MW material might therefore result from either unspecific attachment of sample material to larger particles during sample filtration or (more likely) VLPs (in part) not supporting the chosen electrolyte solution or the nES process and thus particle disintegration to VLP building blocks. Possibly, this low MW material also includes host-cell protein (HCP) contaminations.

As we originally intended to cover the largest possible EM diameter range with our nES GEMMA setup, we opted for a relatively low sheath flow value of air inside the DMA unit of the instrument. In turn, such a low value results in a broad EM diameter analysis range and a bad resolution between peaks. Hence, in a next step, we gradually increased the sheath flow inside the DMA (Figure 2) from 3.5 to 15 Lpm (as the max. possible value of the instrument).

**Figure 2:**
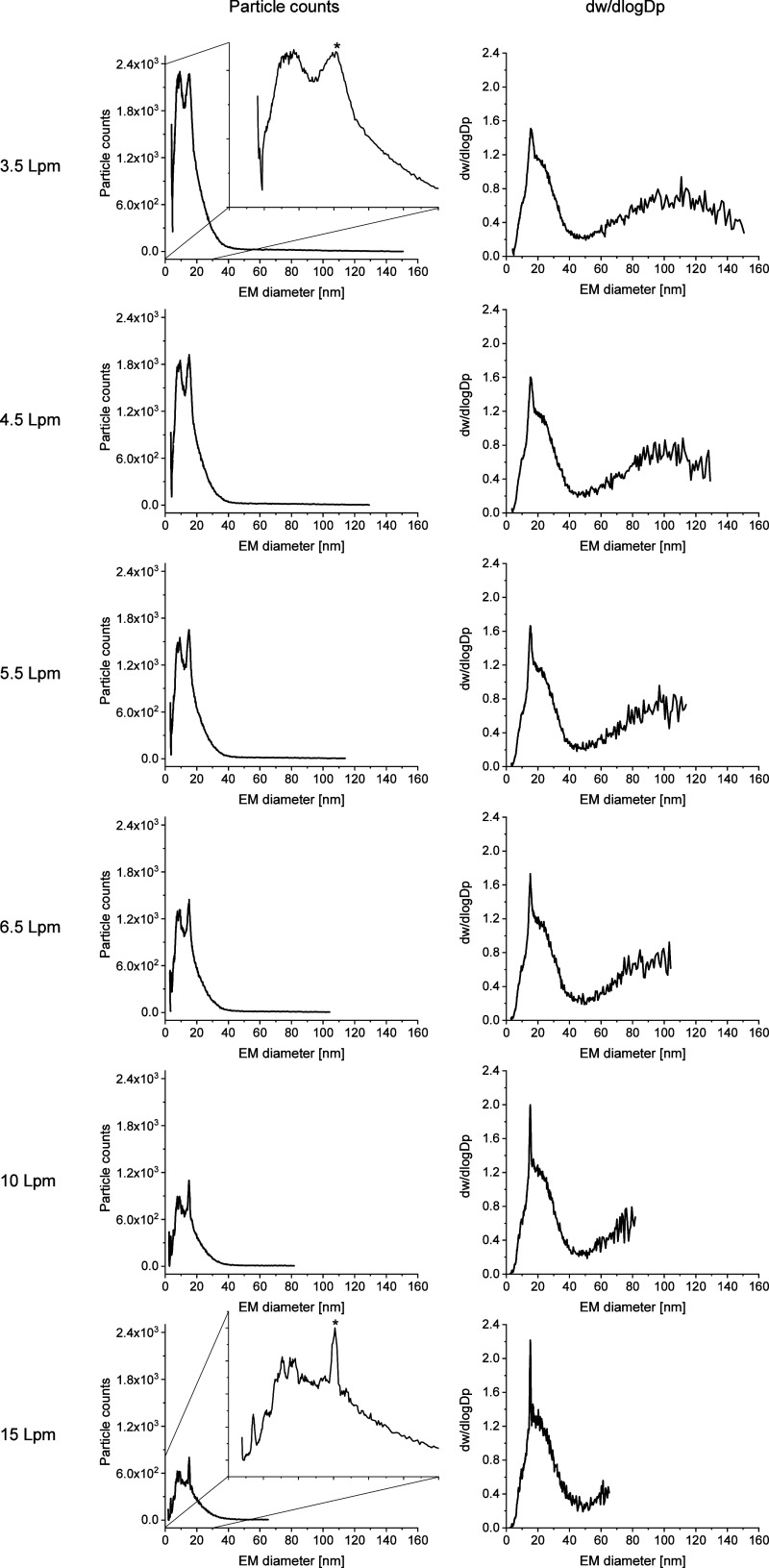
nES GEMMA signals obtained for indicated sheath flow values in the DMA unit of the instrument. Particle count values (left column) and mass-based data (right column) are plotted. With increasing sheath flow values, the scannable EM diameter range diminishes (sizing range is reduced from maximum 150 to 64 nm EM diameter); at the same time, peak resolution in the lower EM diameter range increases. The latter effect can especially be seen in insets for 3.5 Lpm and 15 Lpm settings—for 15 Lpm, the broadness of peaks, e.g., at 15 nm EM diameter (marked by an asterisk), is reduced, which leads to the possibility to identify overall more peaks in the range up to 30 nm EM diameter.

Despite an ultimately reduced overall EM diameter range, we gained resolution for low MW materials in doing so. At a 15 Lpm sheath flow, we were able to discern several peaks in the lower EM diameter range. We reasoned that these probably correspond either to HCPs or to proteins released from SARS-CoV-2 particles upon their collapse (VLP building blocks). Therefore, the MW values of these peaks should correspond to published MW values of N, M, E, and S proteins of SARS-CoV-2.

Based on Uniprot data (retrieved on December 11, 2023), we expected S protein (A0A6G7K2L4) at 141.18 kDa in its monomeric form (trimer in native VLPs), N protein (P0DTC9·NCAP_SARS2) at 45.63 kDa in its monomeric form (homodimer, tetramer, and oligomer in solution), M protein (P0DTC5·VME1_SARS2) at 25.15 kDa in its monomeric form (homomultimer in solution), and E protein (P0DTC4·VEMP_SARS2) at 8.37 kDa in its monomeric form (homopentamer in solution). However, e.g., for S protein, glycosylations are known, influencing the MW value originally given with the Uniprot data.^30^ Due to these post-translational modifications, a higher MW is expected. Indeed, Leadgene Biomedical Inc. reports a signal for S protein at approximately 180 kDa based on Western Blot analysis (https://www.leadgenebio.com/sars-cov-2-virus-like-particles-ldg002pvm.html). Also, Stiving et al. report a higher MW for S protein trimers after glycosylation.^31^ From I^2^MS measurements, they obtained a trimer MW of 540 kDa (i.e., monomer MW of 180 kDa) in comparison to LC–MS-derived data of 520.5 kDa (i.e., monomer MW of 173.5 kDa) or SEC-MALS results (551 kDa S protein trimer, 183.7 kDa monomer^32^).

When fitting several peaks to signals obtained for low MW material in SARS-CoV-2 samples at a 15 Lpm sheath flow in the DMA unit, we obtained peak EM diameter values in a range, which could be correlated to the MW value range of VLP building blocks in monomeric and multimeric forms (application of an EM diameter/MW correlation for proteins, lit.^29^). Figure 3A plots exemplary results from two measurement time points; Table S1 gives an overview of obtained EM diameter values (triplicate measurements, Gauss peaks were fitted via the software Origin) and resulting peak MWs in case a protein-based correlation is used for particle MW calculation. Especially, peak 9 at 9.5 ± 0.1 nm EM diameter (corresponding to roughly 163 kDa on the nES GEMMA-derived protein MW scale) is possibly corresponding to glycosylated SARS-CoV-2 S protein monomers.

**Figure 3:**
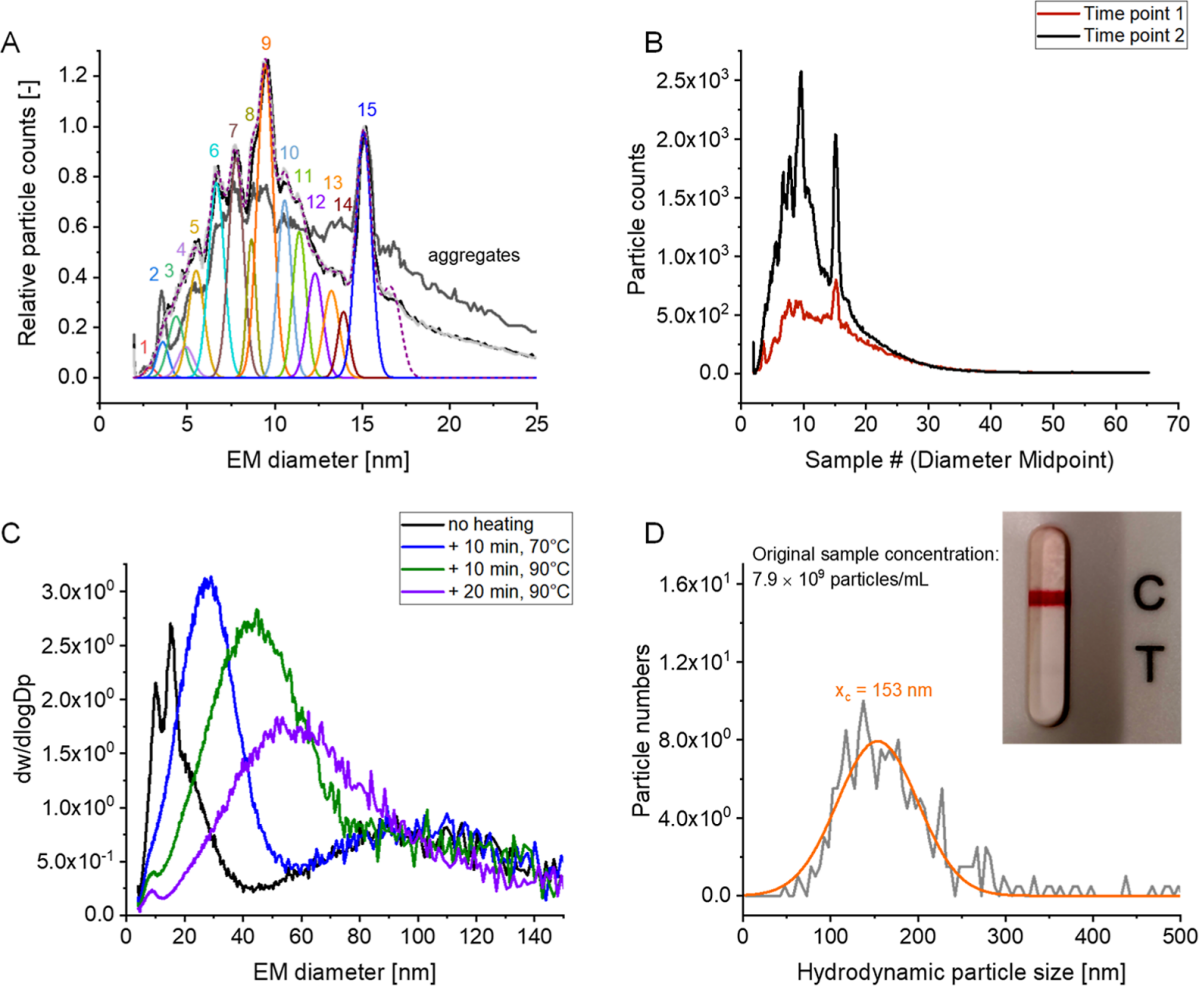
nES GEMMA data for low EM diameter material present in SARS-CoV-2 VLP samples (a sample diluted to 50% [v/v] in ammonium acetate was analyzed). Several Gauss peaks can be fitted to obtained signals (A), which are changing over storage time at 4 °C (B). Additional sample heating for forced VLP degradation leads to unspecific aggregation of low EM diameter material as learned from nES GEMMA spectra (C). NTA (D) shows a decline of particle numbers to 7.9 × 10^9^ particles per mL for the final sample after forced degradation as presented in (C). A reduced NTA particle number (average from two measurements, Gaussian fit in orange to measured data in gray) comes along with a reduced antigenicity as shown in the inset (D). It is of note that for the inset, the image had to be processed (adjustment of saturation parameters) in order to display the second test line at T also in the photograph.

It is of note that data presented in Figure 3A and Table S1 was obtained from overall triplicate measurements at two different time points. Between measurements, samples were stored at 4 °C. As demonstrated in Figure 3B, this storage for approximately 2.5 weeks resulted in increased signals in the lower EM diameter region and several more pronounced peaks. It therefore appears that SARS-CoV-2 VLPs in ammonium acetate show a reduced stability in the chosen electrolyte even at 4 °C and that low EM diameter material indeed originates from SARS-CoV-2 particles upon their collapse (i.e., representing VLP building blocks). In this context, an increase of peak 9 (putatively SARS-CoV-2 S protein monomers) is to be noted. An origin of the low EM diameter material only in HCPs alone is thus not likely but should be targeted in a follow-up study.

### Forced Degradation of SARS-CoV-2 VLPs

Finally, we intended to follow SARS-CoV-2 VLP disintegration under forced conditions. As reported, SARS-CoV-2 is susceptible to heating.^33–35^ Therefore, we heated a corresponding sample successively to 70 and 90 °C, respectively. Heating of the final sample was for 10 min to 70 °C, followed by 10 min heating to 90 °C and 20 min to 90 °C. We anticipated an increase of low EM diameter material with a concomitant reduction of higher EM diameter signals. As depicted in Figure 3C, this procedure led to decrease of low EM diameter material, other than expected. Instead, seemingly unspecific aggregation of VLP building blocks occurred leading to a heterogeneous entity at approximately 30 nm EM diameter. It is of note that with increased sample heating, the apex of this new peak shifted to larger EM diameters. At the same time, VLP material up to 150 nm EM diameter only slightly diminished. This finding was further supported by NTA measurements showing a decrease in particle numbers to 7.9 × 10^9^ particles per mL (roughly 60% of the value before heating) and antigen testing (Figure 3D). The latter only showed a slight band demonstrating antigenicity, where a response comparable to Figure 1B (middle) was expected. Hence, we reasoned that heating of SARS-CoV-2 samples led to aggregation of free VLP building blocks but only slight VLP decomposition possibly due to the absence of genomic material within bionanoparticles which otherwise might have had additionally influenced VLP degradation.

## Conclusion

With our article, we are able for the first time to demonstrate gas-phase electrophoresis of SARS-CoV-2 VLPs. Besides a broad signal which we attributed to intact bionanoparticles in the size range of 50–150 nm EM diameter in accordance with NTA data, we also detected smaller-sized sample components up to 30 nm EM diameter. Fitting of Gauss peaks in this EM diameter range and application of a protein-based EM diameter/MW correlation enabled us to estimate the MW values of corresponding species. These MW values lie in a range as expected for SARS-CoV-2 VLP building blocks. This fact together with the observation that low EM diameter peaks increased with increased sample storage time at 4 °C led us to believe that these species indeed indicate VLP disintegration. Also, HCPs might also contribute to these signals. Lastly, we demonstrated the ability of gas-phase electrophoresis on an nES GEMMA instrument to follow a sample’s response to forced degradation. In doing so, we opted for sample heating to different temperatures and incubation times, seemingly leading to unspecific aggregation of VLP building blocks. At the same time, the peak assigned to intact VLPs only slightly diminished. Ultimately, the unspecific aggregates approached VLP particles in size. Therefore, a careful characterization of SARS-CoV-2 VLP containing samples prior in-depth analysis, e.g., via electron microscopy, seems inevitable in order not to mistakenly account these unspecific aggregates for native VLP complexes. To conclude, despite many still open questions in the characterization of SARS-CoV-2 VLP material, it is our belief that nES GEMMA analysis is a suitable, robust method for the characterization of such bionanomaterials.

## Supplementary Material



## Notes

Table relating details to fitted Gauss peaks from Figure 3 of the main manuscript (PDF)
