# Supplementary material for: Gas-Phase Electrophoresis (nES GEMMA Instrumentation) of SARS-CoV-2-Based Virus-like Particles
Source: ACS Omega. 2025 Nov 12;10(46):55525–32. doi: 10.1021/acsomega.5c06369 (PMC12658705; doi:10.1021/acsomega.5c06369)
Supplement: Supplementary file 1 [file ao5c06369_si_001.pdf]

SUPPLEMENTARY INFORMATION

Gas-phase electrophoresis (nES GEMMA instrumentation) of SARS-CoV-2 based virus-like particles

Victor U. Weiss*, Martina Marchetti-Deschmann

Institute of Chemical Technologies and Analytics, TU Wien, Vienna, Austria

Corresponding author (*): Victor U. Weiss

Institute of Chemical Technologies and Analytics

TU Wien (Vienna University of Technology)

Getreidemarkt 9/164 CTA

1060 Vienna

Austria

Email: victor.weiss@tuwien.ac.at

Phone: 0043 1 58801 151611

The supplement contains a table relating details to fitted Gauss peaks from Figure 3 of the main manuscript.

Supplementary Table 1

List of fitted peaks in Figure 3 and calculated MW values based on a protein EM diameter / MW correlation. This valued y (MW in kDa) = 0.11851 × EMD (nm) ^ 3.20776 and was based on triplicate measurements from Carbonic Anhydrase, Ovalbumin, Bovine Serum Albumin and β-galactosidase. EM diameter averages and STDEV values are from triplicate measurements, if not indicated by an asterisk. For the latter case, n=2 measurements were used. The MW of peak 1 was not calculated due to detection limits of the instrument in the corresponding EM diameter range.

Peak ID	Average EM diameters [nm]	STDEV of EM diameters [nm]	Estimated MW [kDa]
1	2.85	0.05	n.d.
2*	3.56	0.03	7
3	4.26	0.14	12
4*	4.97	0.08	20
5	5.54	0.06	29
6	6.69	0.02	53
7	7.79	0.07	86
8	8.75	0.07	125
9	9.51	0.06	163
10	10.53	0.11	226
11	11.41	0.07	292
12	12.29	0.03	370
13*	13.21	0.03	467
14	13.93	0.10	554
15	15.13	0.05	722
